# Dual-Band Filter and Diplexer Design Using Extremely Miniaturized Substrate-Integrated Coaxial Cavity

**DOI:** 10.3390/s25092921

**Published:** 2025-05-05

**Authors:** Chun-Ming Hung, Ci-Fang Jheng, Keh-Yi Lee, Chung-I G. Hsu, Min-Hua Ho

**Affiliations:** 1 Universal Microwave Technology Inc., Keelung 206, Taiwan; alan_hung@umt-tw.com; 2 Department of Electronic Engineering, National Changhua University of Education, Changhua 500, Taiwan; m1253004@mail.ncue.edu.tw; 3 Department of Electrical Engineering, Chinese Culture University, Taipei 111, Taiwan; kyleentu@ms52.hinet.net; 4 Department of Electrical Engineering, National Yunlin University of Science and Technology, Yunlin 640, Taiwan; cghsu@yuntech.edu.tw

**Keywords:** dual-band filter (DBF), diplexer, substrate-integrated waveguide (SIW), substrate-integrated coaxial cavity (SICC), circuit miniaturization

## Abstract

**Highlights:**

We utilize printed circuit board technology to develop a substantially miniaturized substrate-integrated coaxial cavity (SICC) resonator, achieving a significant size reduction as the circuit occupies a mere 2.1% of the area of its substrate-integrated waveguide cavity counterpart when operating at the same frequency. For the first time, these miniaturized SICC resonators have been successfully incorporated into the design of a dual-band filter and a diplexer, demonstrating a considerable performance improvement.

**Abstract:**

This paper presents the design of a dual-band filter and a diplexer using an extremely miniaturized substrate-integrated coaxial cavity (SICC) structure. The presented dual-band filter can function as a front-end circuit block connected to 5G antennae, enabling dual-passband operation for 5G applications. The diplexer is designed for use in 5G communication systems, positioned after the 5G antennae to facilitate the switching of transmitting (Tx) and receiving (Rx) signals between the Tx and Rx terminals. The main contribution of this work is the development of a highly miniaturized substrate-integrated coaxial cavity (SICC) to design a dual-band filter (DBF) and a diplexer. The circuit area of the proposed dual-frequency SICC is a mere 2.1% of its conventional substrate-integrated waveguide (SIW) cavity counterpart when operating at the same frequency. A dual-band filter and a diplexer are realized using two and three highly miniaturized SICC resonators, respectively. The dual-band filter is designed to have a transmission zero on each passband side to enhance signal selectively. At most in-band frequencies, the isolation between the diplexer’s channel bands exceeds 20 dB. A sample dual-band filter and diplexer have been fabricated for experimental validation, demonstrating excellent agreement between the measured and simulated data. To the best of the authors’ knowledge, the designed dual-band filter and diplexer achieve the highest circuit area efficiency within the categories of dual-band SIW cavity filters and diplexers.

## 1. Introduction

Dual-band filters and diplexers that utilize substrate-integrated waveguide (SIW) technology have attracted the attention of many researchers. The employed SIW structure was first introduced by Zaki et al. [1]. Subsequently, Fuji et al. [2] and Deslandes and Wu [3] reported similar structures under different nomenclatures. The SIW possesses several advantages, including a higher quality factor and enhanced noise immunity, which are not present in other planar structures such as microstrip lines, coplanar waveguides, and slotlines. However, the size of SIW filters is considered excessively large for modern communication systems, particularly when operating at half-wavelength frequencies. Numerous studies have reported various methodologies aimed at reducing the circuit size of SIWs [4,5,6,7,8,9,10,11,12,13,14,15,16,17,18,19,20]. Notably, references [4,5,6] utilize half-mode and folded SIW structures, respectively, reducing the circuit area by a factor of two. Introducing the quarter-mode SIW cavity in [7,8,9,10,11] and the eighth-mode SIW cavity in [10,11,12] necessitates only a quarter/one-eighth of the size of a conventional SIW cavity. In [13,14,15], the size miniaturization of the SIW BPF is accomplished by incorporating complementary split-ring resonators (CSRRs), facilitating the dominant propagation mode to operate at a frequency much lower than the cut-off frequency, thereby effectively decreasing the filter size. However, the filters are susceptible to radiation losses due to the open edges of the SIW and the slots of the CSRR, limiting their applicability to low microwave frequency ranges. In [16], inserting a dielectric rod within the SIW cavity excites the TM_nm0_ mode, resulting in a 33% reduction in size. A more substantial size reduction of 60% is reported in [17] by implementing a concave–convex cavity filter. Recently, an exceptional miniaturization design was introduced in [18], achieving a 93% reduction in circuit size by loading the quarter-mode SIW cavity with a complicated capacitive structure. Furthermore, the evanescent-mode SIW cavities, also referred to as coaxial resonators or embedded coaxial SIWs, in [19,20,21] demonstrate even greater size reductions, with circuit area reductions exceeding 96% for a single SIW cavity resonator.

In recent years, bandpass filters (BPFs) with dual-band functions have received immense demand since wireless communication systems exhibit a trend toward a multichannel operation. Numerous substrate-integrated waveguide (SIW) BPFs [22,23,24,25,26,27] have been developed to meet the dual-passband requirements. Among these, a compact dual-band BPF was designed in [22], utilizing multiple SIW cavities arranged in a vertically stacked configuration. However, the slot incurs excessive losses when utilized to subdue common-mode signals at high frequencies. The SIW cavity BPF of dual-mode response presented in [23] utilizes two metallic rods of differing sizes, each capable of independently tuning its associated resonance frequency, thereby facilitating the formation of two independently controlled passbands. In [24], a single circular SIW cavity operating under multiple transverse magnetic (TM) modes is utilized for dual-band filter design. The independent control of the dual-band frequencies is ingeniously achieved by perturbing the TM modes using additional via-holes in conjunction with the slots embedded in the cavity wall. In [25], the dual-band response is realized by incorporating complementary split-ring resonators (CSRRs) embedded in the SIW cavity walls, leveraging degenerate-mode resonance. Each CSRR acts as an additional resonance node extending from the cavity, creating transmission zeros (TZs) on either side of passband edges. Furthermore, as noted in [26], an E-slot embedded in the SIW cavity renders the latter a dual-band function, with the resonances of the E-slot’s even- and odd-mode resonances dominating the passbands’ frequencies. Moreover, the half-mode rectangular SIW cavity was initially utilized in dual-band filter applications, as discussed in reference [27], allowing for a significant separation between the two passbands. Overall, the aforementioned SIW cavities in [22,23,24,25,26,27] are relatively large and, as such, are not well-suited for commercial microwave systems that necessitate circuit miniaturization.

The dual-band SIW cavity BPFs are also applicable in the design of diplexers, which facilitate the routing of signals at two distinct frequencies to separate ports. Recent studies have reported several innovative diplexers utilizing SIW cavity designs [28,29,30,31,32,33]. For instance, the hexagonal SIW cavity diplexer described in [28] employs cavities that operate under perturbed degenerate-mode resonance for the transmitting (Tx) and receiving (Rx) filters. However, a notable limitation of this design is the relatively large size of the cavities utilized. In [29], a diplexer is constructed using two cavities that operate under perturbed dual-mode resonances. Each cavity generates a transmission zero (TZ) positioned within the passband of the opposing cavity filter to enhance isolation. Although this design incorporates only two SIW cavities, it necessitates a substantial SIW T-junction to connect the common port to both the Tx and Rx cavities. The work presented in [30] introduces a sophisticated diplexer design that features frequency-tuning capabilities and accommodates various port configurations, including single-ended and balanced forms. Frequency tuning is achieved by incorporating piezoelectric disks into the cavity, which load the cavity with distinct capacitances. A compact SIW diplexer composed of combined single- and dual-mode cavities is documented in [31]. This configuration enables the two duplexing bands to have a flexible bandwidth ratio; however, it requires a considerable circuit area due to the necessity of multiple SIW cavities. In [32], integrating a microstrip filter within three air-filled circular SIW cavities successfully facilitates the separation of two duplexing bands. The microstrip filter routes the low-frequency signal in this configuration, while the air-filled SIW cavities channel the high-frequency millimeter-wave signal. Reference [33] uses a field perturbation on the SIW’s multi-mode to create the filter’s multiple passband functions. The aforementioned SIW multi-band BFSs and diplexers exhibit a relatively large circuit area, which may limit their suitability for commercial applications.

In our previous study, we investigated a miniaturized substrate-integrated coaxial cavity (SICC) structure, which has been effectively utilized in the designs of size-reduced single-band [20,34], dual-band [35], tri-band [36], and quad-band [37] BPFs, as well as a diplexer [35]. While SICC technology has achieved and demonstrated the highest circuit area efficiency for substrate-integrated waveguide (SIW)-related cavities, ongoing advancements in the reduction of SIW cavity size are still being pursued. Reference [38] illustrates a further size reduction technique for the SICC structure by enhancing the SICC’s loading capacitance through a split coplanar waveguide ring (SCR). However, the current advancements pertain solely to single-band filters (BPFs). Consequently, there is a need for further research and study into dual-band BPFs and diplexers that utilize this ultra-miniaturized SICC technology. Hence, this paper presents the design of a dual-band BPF and diplexer employing the extremely size-reduced SICC structure. Sample circuits have been fabricated for experimental validation, revealing good agreement between the measured and simulated data.

## 2. Implementation of Miniaturized Substrate-Integrated Coaxial Cavity

**Figure 1:**
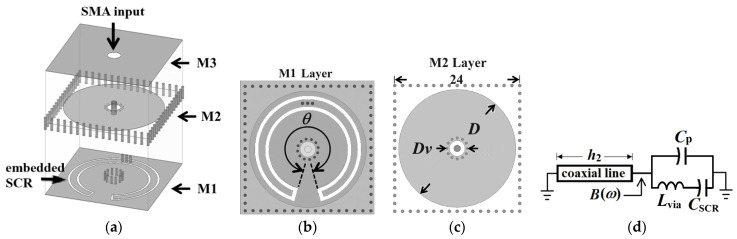
Layouts of the extremely miniaturized substrate-integrated coaxial cavity: (**a**) 3D view, (**b**) bottom (M1) layer embedded with the split CPW ring (with the subtended angle *θ*), (**c**) middle (M2) layer, and (**d**) the equivalent circuit model.

The proposed extremely miniaturized substrate-integrated coaxial cavity (SICC) and its corresponding equivalent circuit model are illustrated in Figure 1. This SICC consists of two tightly stacked RT/Duroid 5880 substrates (*ε_r_* = 2.2 and tan*δ* = 0.0009) with thicknesses *h*_1_ = 0.254 mm for the top substrate and *h*_2_ = 1.57 mm for the bottom substrate. Additionally, the configuration includes three metal sheets, each with a thickness of 35 μm. A 0.08 mm thick prepreg (PP) layer, with *ε_r_* = 4 and tan*δ* = 0.013, is positioned between the middle metal layer and the top substrate to facilitate substrate binding. The equivalent circuit model depicted in Figure 1d consists of a short-circuited coaxial line, with a length of *h*_2_, which is loaded with capacitances *C*_p_, *C*_SCR_, and an inductance *L*_via_. The capacitance *C*_p_, approximated by *C*_p_ ≈ 0.25*πεD*^2^(1/*h*_1_ + 1/*h*_2_), represents the combined capacitance between the circular patch of diameter *D* (in the M2 layer) and the top and bottom walls. The term *C*_SCR_ stands for the capacitance between the center strip of the embedded split coplanar waveguide ring (SCR) and the bottom ground plane. At the same time, *L*_via_ denotes the inductance associated with the three blind via-holes that connect the embedded SCR to the circular patch. It is noteworthy that, as referenced in [39], *C*_SCR_ can be evaluated as follows:

(1a) $$C_{\mathrm{SCR}}=\left(2\varepsilon_{0}(\varepsilon_{r}-1)\frac{K(k_{1})}{K(k^{\prime })}+4\varepsilon_{0}\frac{K(k_{0})}{K(k_{0}^{\prime })}\right)l_{\mathrm{SCR}}$$

(1b) $$k_{0}=S/(S+2W),\;k_{0}^{\prime }=\sqrt{1-k_{0}^{2}}$$

(1c) $$k_{1}=\frac{\sinh(\pi S/4h_{2})}{\sinh\left[\pi (S+2W)/4h_{2}\right]},\;k_{1}^{\prime }=\sqrt{1-k_{1}^{2}},$$

where *K*(∙) is the complete elliptic integral of the first kind, while the parameters *S* and *W* denote the strip and slot width of the embedded SCR, respectively. Moreover, $\varepsilon_{0}$ is the permittivity in vacuum, and *ε_r_* is the substrate’s dielectric constant. Additionally, *l*_SCR_ refers to the mean arc length of the SCR, measured in millimeters (mm). Based on the equivalent circuit model, the total susceptance *B*(*ω*) between the circular patch and the ground, as illustrated in Figure 1d, is expressed as follows:

(2) $$B(\omega )=\omega \;\left(\frac{C_{\mathrm{SCR}}}{1-\omega^{2}L_{\mathrm{via}}C_{\mathrm{SCR}}}+C_{p}\right)-Y_{0}\cot\beta h_{2}$$

where the phase constant is denoted by *β*, with *c*_0_ representing the speed of light in vacuum and *Y*_0_ indicating the characteristic admittance of the short-circuited coaxial transmission line formed by the blind via-ring fence of diameter *D_v_* and the four thru-via side walls of the square cavity. For convenience, the frequency-dependent expression in the parentheses of Equation (2) is referred to as the effective loading capacitance *C*_eff_. This expression is applicable when the operating frequency is much lower than the series resonance frequency of the *L*_via_-*C*_SCR_ branch.

In a conventional square SIW cavity, the fundamental TE_101_ exhibits a resonance frequency denoted as *f*_0_. In addition to this fundamental mode, the proposed SICC resonates as a capacitively loaded coaxial-line mode oriented along the normal direction of the substrate, characterized by a resonance frequency of *f_r_*. Since the lower substrate has a small electric thickness *βh*_2_ around the desired operating frequencies, the approximation cot(*βh*_2_)≈1/*βh*_2_ can be made, causing the condition *B*(*ω*) = 0 in Equation (2) to yield the resonance frequency *f_r_* as [20].

(3) $$f_{r}=\frac{1}{2\pi }{\left[\frac{Y_{0}c_{0}}{C_{\mathrm{eff}}\sqrt{\varepsilon_{r}}h_{2}}\right]}^{1/2}$$

In contrast to our previous works in [34,35,36,37], which incorporated only a single capacitance within the cavity, the proposed SICC presented in this paper significantly enhances the overall capacitance. This enhancement is attributed to integrating an additional capacitance, which is facilitated by the SCR embedded in the M3 layer, as illustrated in Figure 1a. The supplementary capacitance can now be integrated with the capacitance *C*_p_ through three connection via-holes to form the enhanced effective loading capacitance *C*_eff_. This configuration not only enhances the loading capacitance of the SICC but also leads to a further downshift in the resonance frequency. In this paper, the side dimension of the square SICC and the conventional square SIW cavity counterpart is fixed at 24 mm. The resonance frequency of the conventional square SIW cavity is *f*_0_ = 5.96 GHz. The frequency ratios *f_r_*/*f*_0_ and *C*_eff_, with their values calculated as functions of the SCR’s subtended angle (*θ*) in the SICC, are omitted here and can be found in [38]. It should be noted that the SICC area is proportional to (*f_r_*/*f*_0_)^2^. The outcome of *f_r_*/*f*_0_ < 1 indicates that the circuit size has been reduced, and all values of *f_r_*/*f*_0_ obtained in this paper are less than 0.15.

**Figure 2:**
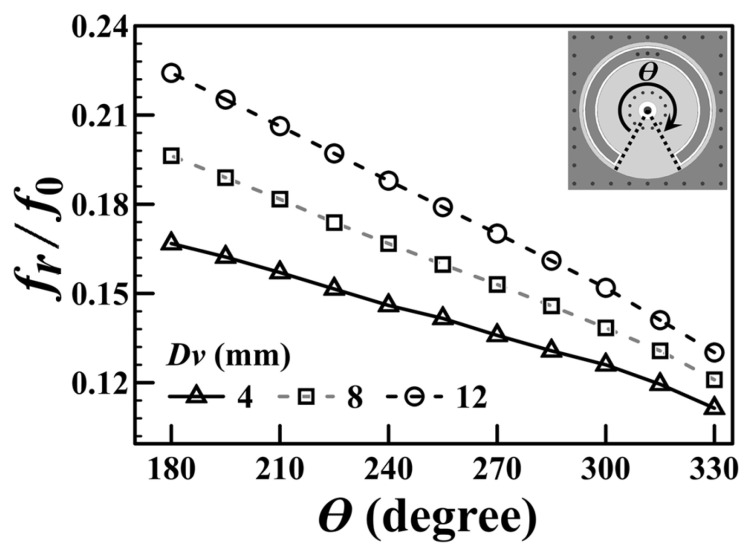
Normalized resonance frequency (*f_r_*/*f*_0_) versus the SCR’s subtended angle (*θ*) for several via-ring-fence diameters (*D_v_*).

**Figure 3:**
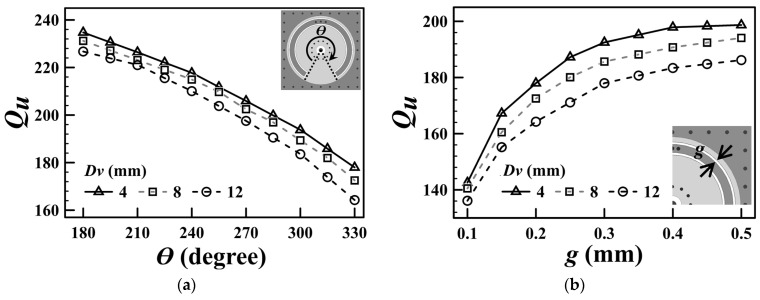
Simulated *Q_u_* values as a function of (**a**) SCR’s subtended angle (*θ*) and (**b**) the SCR’s slot gap dimension (*g*) for several values of *D_v_*.

Figure 2 illustrates the normalized resonance frequency of the SICC depicted in Figure 1 as a function of the subtended angle (*θ*) of the embedded SCR. The figure presents the simulated frequency ratio (*f_r_*/*f*_0_) against the SCR’s subtended angle (*θ*) for various diameters of the via-ring fence (*D_v_*), with the outer diameter of the SCR fixed at 20.8 mm. The data indicate that an increase in the subtended angle (*θ*) corresponds to a decrease in the resonance frequency (*f_r_*). This phenomenon can be attributed to the fact that a larger value of *θ* increases the capacitance *C*_SCR_ for the specified dimensions of the SCR. However, the drawback of the resonance frequency downshift is a decrease in the *Q_u_* value, as seen in Figure 3. This is because the larger the value of *θ*, the larger the conduction and radiation losses due to the longer aperture in the cavity’s bottom wall. Furthermore, because the blind via-ring fence in the cavity’s lower substrate region emulates the coaxial line’s center conductor, a smaller via-ring-fence diameter (*D_v_*) yields a smaller characteristic admittance (*Y*_0_), resulting in a lower *f_r_*, as is evident from Figure 2 and Equation (3).

As previously mentioned, an increase in the subtended angle leads to a decrease in the unloaded *Q_u_* value. Figure 3a illustrates the relationship between the unloaded quality factor *Q_u_* and the SCR’s subtended angle. Additionally, Figure 3b presents the variation of *Q_u_* as a function of the SCR’s bilateral slot gap dimension (*g*), revealing that a reduced gap dimension leads to a smaller *Q_u_* value. This phenomenon can be attributed to the greater conduction currents at the edges of the smaller gap, which result in increased conduction losses. Notably, *Q_u_* is relatively insensitive to changes in the diameter (*D_v_*) of the via-ring fence.

## 3. Miniaturized Dual-Band SICC BPF

**Figure 4:**
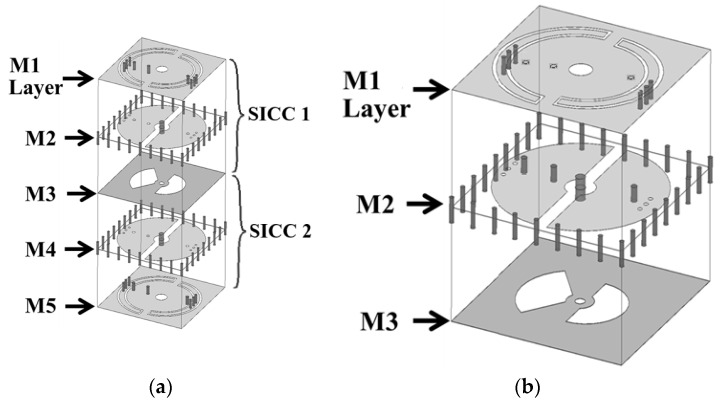
The proposed extremely miniaturized dual-band SICC BPF structure: (**a**) 3D view and (**b**) zoom-in of SICC unit.

**Figure 5:**
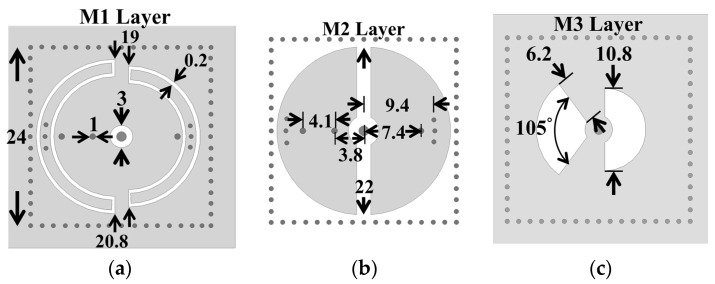
The layouts and circuit dimensions of the BPF in Figure 4b: (**a**) M1, (**b**) M2, and (**c**) M3 layers.

A sample miniaturized dual-band SIW cavity BPF is presented in this session. Figure 4 shows the structure of the proposed extremely miniaturized dual-band SICC BPF. The proposed dual-band BPF is composed of two SICCs (Figure 1) in a vertically stacked form, as shown in Figure 4a. Each cavity comprises two tightly bonded RT/Duroid 5880 substrates of 1.57 (top substrate) and 0.254 mm (bottom substrate) thicknesses. Notably, one cavity is inverted and positioned atop the other, resulting in a total of four substrate layers (five metal layers) for the filter. The feeding structure consists of a sub-miniature version A (SMA) connector on the cavity’s top wall. The SMA probe extends through the cavity and connects to the bottom wall, establishing a short-circuit feed. Figure 5 gives the layouts of the three metal layers (see Figure 4b) and their circuit dimensions (in mm). The middle (M2) layer exhibits two semicircular patches, each of which is connected to the M1 layer of the cavity through one (for the right-side patch) or two extra blind via-holes (for the left-side patch). The former is associated with the low-frequency resonance, and the latter with the high-frequency resonance. In other words, one square cavity houses two resonators. For convenience, we can call each of the two resonators in the same cavity a half-mode coaxial resonator. The low-frequency resonance predominantly exists in the right half of the cavity, whereas the high-frequency one exists in the left half of the cavity. When the dimensions of the semicircular patch are fixed, the resonance frequency of each half-mode coaxial resonator is predominantly influenced by the dimensions of the associated SCR and the extra blind shorting via-holes positioned at appropriate locations. In Figure 1, the circular patch and the bottom cavity wall are connected using the blind via-ring fence of diameter *D*_v_. This blind via-ring fence, in conjunction with the surrounding cavity, forms a short-circuited coaxial line. Under the condition that the lower substrate has a small electric thickness, the equivalent lumped element looking into the short-circuited coaxial line from the circular patch is an inductance. In the half-mode coaxial resonator, the extra blind shorting via-holes connect the semicircular patch to the bottom cavity wall, and they play the same role as the blind via-ring fence in Figure 1.

The equations presented in Section 2 may no longer accurately evaluate the resonance frequencies for the proposed structure of two resonators within one cavity. However, the trend of the resonance frequencies is similar to that of the complete SICC case. Nevertheless, an empirical approach combined with an L-C circuit model, as utilized in [35,36], is used to determine the resonance frequency of each half-mode coaxial resonator. This method involves fixing the loaded capacitances (i.e., fixing the size of the semicircular patch and the SCR) while varying the number and positions of the extra shorting vias that connect the semicircular patch to the bottom wall of the cavity, effectively controlling the inductance value for each L-C resonance. Intuitively, the right-side half-mode coaxial resonator with only one extra blind via-hole can be regarded as having a larger effective inductance in the L-C circuit model than the left-side one with two extra blind via-holes, causing the former to have a lower resonance frequency.

**Figure 6:**
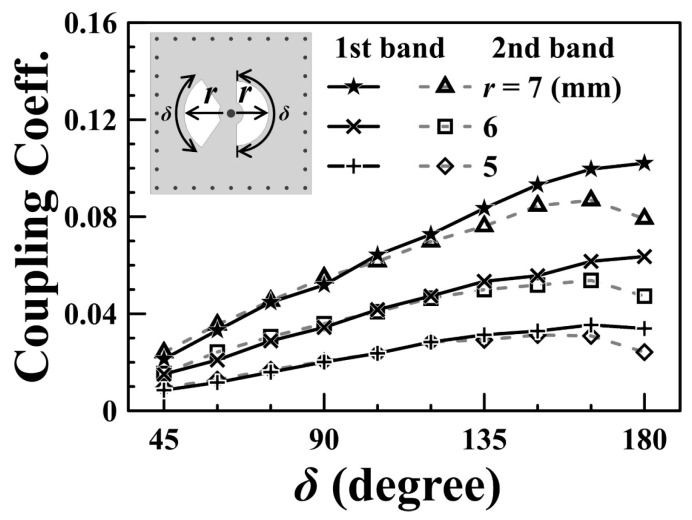
Coupling coefficient versus the slot’s subtended angle for various slot radii.

The coupling between the upper and lower SICCs relies on the two fan-shaped slots embedded in the common wall (the M3 layer in Figure 4a and Figure 5c) of the two SICCs. The left-side slot depicted in Figure 5c is smaller than the right-side one, which is larger; this configuration allows the left-side slot to suitably function for the high-end passband, while the right-side slot operates better at the low-end passband. The nature of this coupling is classified as an electrical coupling, resulting in the patches on either side of the coupling slot exhibiting opposite electric potentials. The calculated coupling coefficients for the low-end (first) and the high-end (second) passbands as functions of the subtended angle (*δ*) of the coupling slots are presented in Figure 6 for various radius values (*r*) of fan-shaped slots.

**Figure 7:**
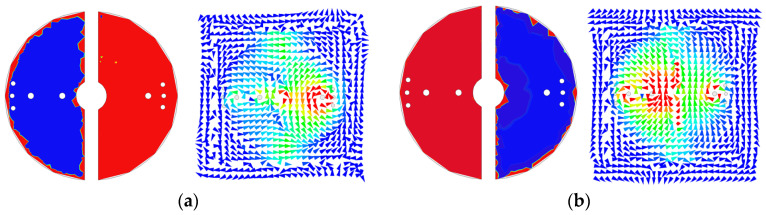
The distributions of electric field and vector magnetic field at the (**a**) first and (**b**) second passbands.

Figure 7 presents the distributions of the electric field (E-field) and the vector magnetic field (H-field) for the first and second passbands. The E-field is calculated on the upper surface of the metal patch, while the H-field is simulated beneath the surface of the metal patch, to which the extra blind shorting via-holes are connected. In the first passband, as illustrated in Figure 7a, the right semicircular patch exhibits a resonance predominating at low frequencies. Furthermore, the H-field is also significantly stronger in the right-side region. In contrast, the second passband reveals a greater E-field strength on the left semicircular patch, accompanied by a more enhanced H-field also in the left cavity. The H-field distribution surrounding the two shorting via-holes of the left patch shows that the H-field encompasses both via-holes. This observation implies that the inductances created by the blind shorting via-holes are arranged in a parallel configuration, resulting in a total inductance less than that of a single blind shorting via-hole. Consequently, this smaller inductance leads to a higher resonance frequency.

As observed from Figure 7a, when the right-side half-mode coaxial resonator resonates at the first passband, the left-side half-mode coaxial resonator is not entirely silent. Similarly, from Figure 7b, when the left-side half-mode coaxial resonator resonates at the second passband, there are still fields in the right-side half-mode coaxial resonator. This is because the left- and right-side half-mode coaxial resonators are not entirely isolated. Adjustment of the structural dimensions of the left-side (right-side) half-mode coaxial resonator for tuning the lower (higher) resonance frequency would affect the higher (lower) resonance frequency. Hence, we cannot claim that the two resonance frequencies can be independently controlled. Fortunately, the field strengths in the left- and right-side half-mode coaxial resonators are pretty distinct, and structural dimensions can easily be adjusted to obtain the two desired resonance frequencies.

**Figure 8:**
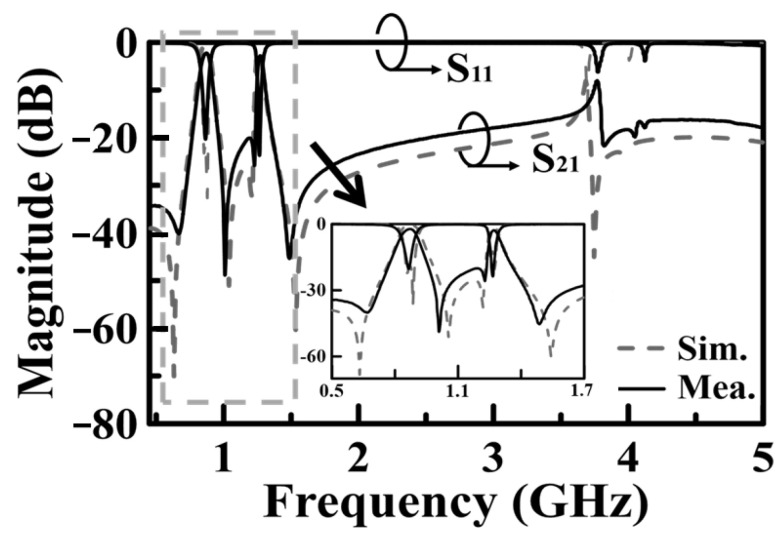
Measured and simulated frequency responses for the BPF of Figure 4.

**Figure 9:**
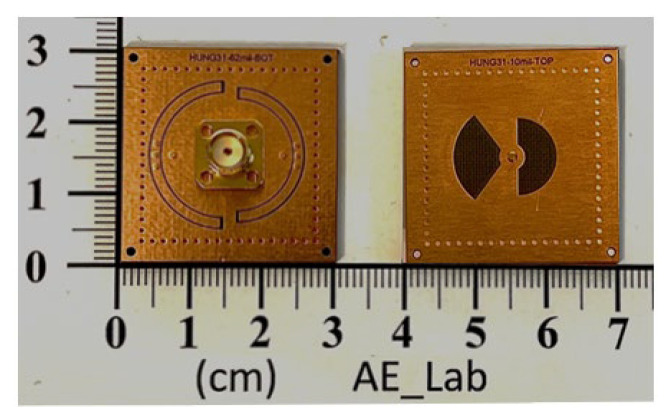
Photos of the experimental circuit, its top and coupling slots views.

The frequency responses, both measured and simulated, are presented in Figure 8, with the inset illustrating the zoomed-in passband responses. The coupling mechanism is primarily dominated by the electric field (E-field) within the passband regions, transitioning to dominance by the magnetic field (H-field) in the stopband region. A transmission zero (TZ) is generated during the coupling transition from E-field to H-field dominance and vice versa, forming two TZs on either side of each passband. The coupling slot’s axial dimensions (and subtended angle *δ*) are, respectively, 5.4 (*δ* = 180°) and 6.2 mm (*δ* = 105°), corresponding to coupling coefficients of 0.042 and 0.038 for the first and second passbands, respectively. The calculated unloaded *Q_u_* values are 166 and 239 for the cavity operating at the first and second resonance frequencies, respectively. These values are lower than the *Q_u_* value of 680 associated with the conventional SIW cavity counterpart, which depletes every structure within the SIW cavity. The prepreg film used in the printed circuit board (PCB) technology for binding purposes significantly impacts the performance of the proposed circuit due to its lossy nature and its placement over the middle patch layer, where the electric field strength is maximized. When depleting the loss associated with the prepreg film, the *Q_u_* value increases to 282 for the first band and 375 for the second band. The measured and simulated performance parameters observed in Figure 8 are summarized in Table 1, which includes mid-band frequencies, 3DB fractional bandwidths (FBWs), minimum in-band insertion losses (ILs), transmission zeros (TZs), and calculated *Q_u_*. The external quality factor, *Q_e_*, is 12.36 for the first band and 25.51 for the second band. Figure 9 shows the photos of the circuit fabricated for measurement. Table 2 compares our design with several previously referenced SIW-related dual-band filters. It should be noted that the upper stopband bandwidths listed in Table 2 are under the criterion of |S_21_| ≤ –15 dB. Furthermore, the BPF in [24] has the largest electric dimension in the table and is chosen as the reference for size comparison.

## 4. Miniaturized SICC Diplexer

**Figure 10:**
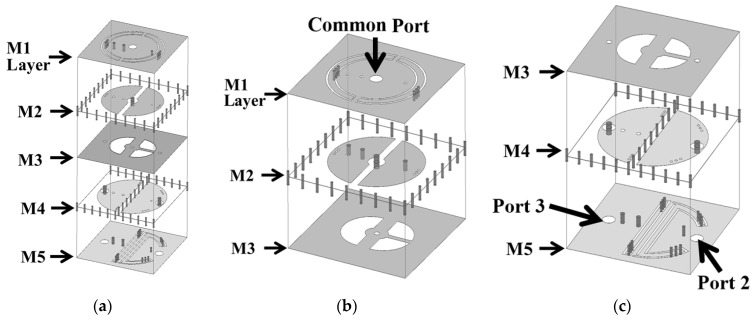
The extremely miniaturized SICC diplexer structure: (**a**) 3D view, (**b**) zoom-in of the common port SICC, and (**c**) zoom-in of the diplexer port cavities.

**Figure 11:**
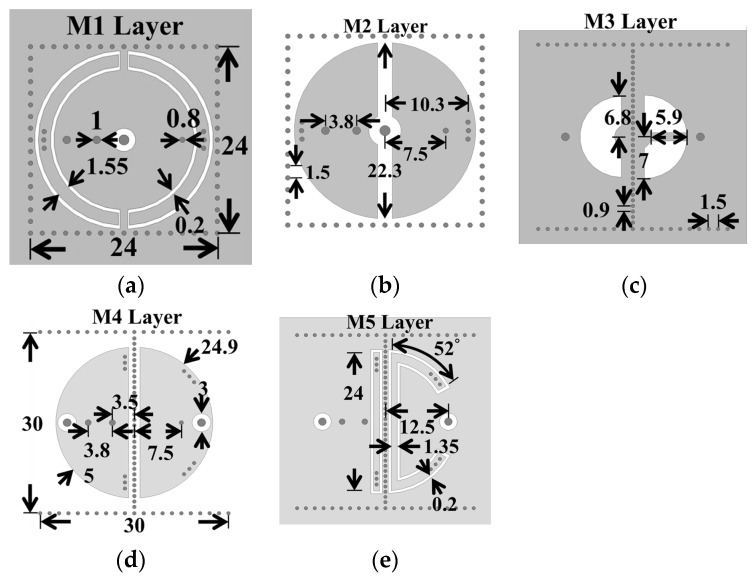
Layouts and circuit dimensions of the metal layers in Figure 10: (**a**) M1, (**b**) M2, (**c**) M3, (**d**) M4, and (**e**) M5 layers.

The 3D view of the proposed miniaturized SICC diplexer is presented in Figure 10, while the circuit layouts and dimensions for each of the metal layers are detailed in Figure 11. The diplexer consists of three SICCs, with the common-port (CP) cavity designed to function in a dual-band operation. The SICCs are fabricated using two RT/Duroid 5880 substrates, with thicknesses of 1.57 mm for the upper substrate and 0.254 mm for the lower substrate. Standard PCB technology is utilized for the diplexer’s fabrication, employing the same prepreg film referenced in Section 2 and Section 3 for binding purposes. The CP cavity incorporates two semicircular patches for dual-band functionality (see Figure 11b). The semicircular patch for the low-band resonance is connected to the cavity’s bottom wall through a single extra blind shorting via-hole, and the one for the high-band resonance is equipped with two extra blind shorting via-holes. Per the methodology outlined in Section 2, each half of the CP cavity has a semicircular SCR of varying size embedded in the M1 layer and three via-holes connecting to the patch to facilitate further frequency downshift. Bisecting a single SICC into two equal-sized half-SICCs results in two duplexing-port (DP) cavities. This bisecting is performed by deploying an array, as illustrated in Figure 11d,e, along the symmetric axis of the cavity, thereby physically separating the two DP cavities. Notably, the half-SICC-formed DP cavity cuts the circuit area in half. The high- and low-frequency signals of the CP cavity are coupled to either of the two DP cavities through a semicircular coupling slot for the high-band signal, or two quarter-circular slots for the low-band signal (see Figure 11c). The port-2 cavity in Figure 10c, featuring a larger SCR and a single extra blind shorting via-hole, is designed to respond to low-band resonance. In contrast, the port-3 cavity is equipped with a straight SCR and two extra blind shorting via-holes, thereby serving the high-band function.

**Figure 12:**
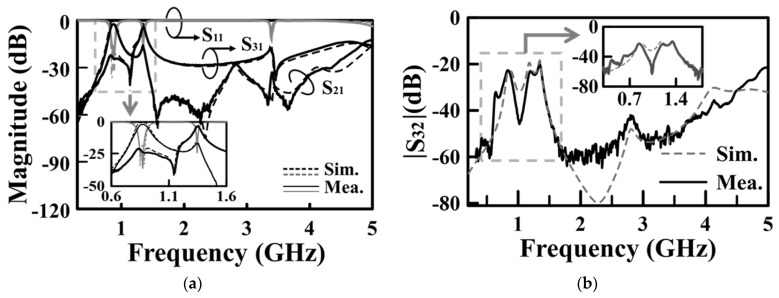
Measured and simulated frequency responses for the SICC diplexer of Figure 11: (**a**) S_11_, S_21_, and S_31_; (**b**) duplexing ports isolation (in terms of |S_32_|).

**Figure 13:**
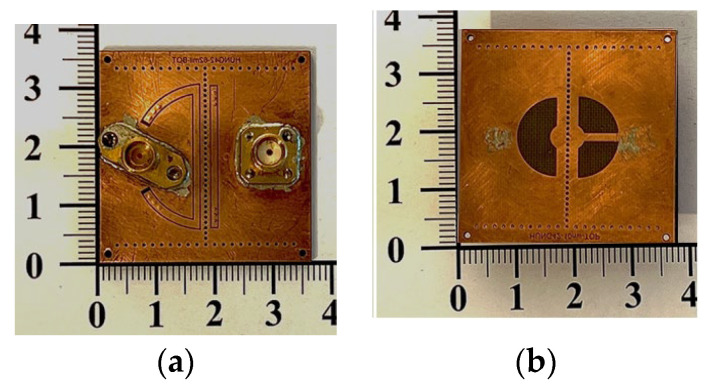
Photos of the experimental circuit in Figure 11: (**a**) duplexing ports and (**b**) coupling slots layer.

Figure 12 shows the measured and simulated frequency responses for the diplexer shown in Figure 11. The measured and simulated performance parameters are summarized in Table 3, which includes mid-band frequencies, FBWs, and in-band minimum ILs. Not listed in Table 3, most measured isolations between the two duplexing ports exceed 20 dB. In Figure 13, we give the photos of the circuit fabricated for measurement. Table 4 compares our design with several previously referenced SIW-related diplexers. This table summarizes the number of substrates used in the circuits, mid-band frequencies, minimum ILs, isolation levels between the duplexing bands, and the circuit area, which is defined as the area of the smallest rectangle capable of accommodating the cavities. Here, the diplexer in [29] is chosen as the reference for size comparison. In conclusion, our diplexer demonstrates a significant circuit area efficiency and considerable duplexing-band isolation. However, the ILs are slightly higher than those observed in other designs. These higher ILs might be owing to the significant losses associated with the prepreg film and the conduction losses resulting from the intensified electric field at the SCRs’ slot edges.

## 5. Conclusions

This paper presents the design, implementation, and verification of a highly miniaturized dual-band substrate-integrated coaxial cavity (SICC) bandpass filter (BPF) and diplexer. The results demonstrate an excellent agreement between the measured and simulated data, affirming the well-structured circuit’s effective performance. The proposed dual-band SICC BPF and diplexer occupy circuit areas of only 0.103 *λ_d_* × 0.103 *λ_d_* and 0.127 *λ_d_* × 0.127 *λ_d_*, respectively. To the best of the authors’ knowledge, the proposed dual-band SICC BPF and diplexer designs have presented the best circuit area efficiency achieved within the dual-band SIW cavity filters and diplexers categories.

## Figures and Tables

**Table 1:** The measured and simulated performance parameters concluded from Figure 8.

	Simulations	Measurements
*f*_1/_*f*_2_ (GHz)	0.87/1.27	0.865/1.275
IL_1_/IL_2_ (dB)	0.74/1.61	1.94/2.51
3DB FBW_1_/FBW_2_ (%)	11.49/4.03	8.09/3.92
Stopband BW (GHz) (|S_21_| ≤ –20 dB)	1.06	1.76
TZs (GHz)	0.63/1.05/1.22/1.54	0.65/1.01/1.23/1.48
*Q_u_*_1__/_ *Q_u_*_2_	166/239	

**Table 2:** The comparison between our design and several referenced dual-band SIW cavity BPFs.

	No. of Layers	f_1_/f_2_ (GHz)	FBW_1_/FBW_2_ (%)	IL_1_/IL_2_ (dB)	USB BW	Size (λ_d_ × λ_d_)	Size Ratio (%)
[10]	2	3.5/5.24	2.73/5.28	1.52/1.65	N/A	1.23 × 1.23	95.3
[23]	3	2.51/5.3	6.8/5.8	1.41/1.88	~0.29 *f*_1_	0.84 × 0.42	22.2
[24]	1	7.71/9.64	5.45/8.1	1.9/1.65	N/A	1.26 × 1.26	100
[25]	1	7.89/8.89	3.4/3.9	1.5/1.9	~0.07 *f*_1_	1.12 × 1.05	74.1
[26] (Figure 4)	1	3.6/6.4	3.3/2.4	1.3/1.8	>0.72 *f*_1_	0.43 × 0.88	23.8
[27] (Figure 6)	1	5.0/7.5	5.46/4.75	1.65/2.25	~0.23 *f*_1_	1.65 × 0.93	96.7
[35] (Figure 2)	4	1.81/2.43	6.06/2.9	1.04/2.3	0.24 *f*_1_	0.21 × 0.21	2.78
This work	4	0.865/1.275	8.09/3.92	1.94/2.51	2.52 *f*_1_	0.103 × 0.103	0.67

USB BW: upper stopband BW with |S_21_| < –15 dB; $\lambda_{d}=c_{0}/(f_{1}\sqrt{\varepsilon_{r}})$, $c_{0}$ is the speed of light.

**Table 3:** The measured and simulated performance parameters concluded from Figure 12.

	Simulations	Measurements
*f*_1/_*f*_2_ (GHz)	0.875/1.35	0.854/1.355
3dB FBW_1_/FBW_2_ (%)	12.57/3.27	10.54/3.69
In-band isolations (dB)	19.2–23.4	19.5–23.01
IL_1_/IL_2_ (dB)	0.62/2.05	1.63/2.89
*Q_u_*_1_/*Q_u_*_2_	126/275	

**Table 4:** The comparison of our diplexer design with the previously referenced SIW diplexers.

	No. of Substrates	f_1_/f_2_ (GHz)	IL_1_/IL_2_ (dB)	IBI (dB)	Size (λ_d_ × λ_d_)	Size Ratio (%)
[28]	1	9.49/9.91	1.42/1.38	N/A	Large	>>100
[29] (Figure 9)	1	9.94/10.95	1.8/2.5	>50	4.1 × 1.57	100
[30] (Figure 10)	3	2.07/2.71	1.1/1.6	>36	0.39 × 0.38	2.3
[31]	1	12/14	1.34/1.41	>27	1.99 × 1.39	43.0
[32]	3	2.35/29.87	0.94/1.32	>22	0.46 × 0.34	2.43
[35] (Figure 13)	4	1.63/1.96	2.5/2.53	>34	0.19 × 0.19	0.56
This work	4	0.854/1.355	1.63/2.89	>19.5	0.127 × 0.127	0.25

IBI: in-band isolation; $\lambda_{d}=c_{0}/(f_{1}\sqrt{\varepsilon_{r}})$.

## Data Availability

The data presented in this study are available on request from the corresponding author.
